# Astragaloside IV improves pulmonary arterial hypertension by increasing the expression of CCN1 and activating the ERK1/2 pathway

**DOI:** 10.1111/jcmm.17681

**Published:** 2023-02-10

**Authors:** Yu Liu, Bai‐Lin Tang, Mei‐Li Lu, Hong‐Xin Wang

**Affiliations:** ^1^ Key Laboratory of Cardiovascular and Cerebrovascular Drug Research of Liaoning Province Jinzhou Medical University Jinzhou China

**Keywords:** astragaloside IV, CCN1, ERK1/2, proteomics, pulmonary arterial hypertension

## Abstract

The aim of the present study was to investigate the underlying mechanism of AS‐IV and CCN1 in PAH and to evaluate whether the protective effect of AS‐IV against PAH is associated with CCN1 and its related signalling pathway. In vivo, male SD rats were intraperitoneally injected with monocrotaline (MCT, 60 mg/kg) or exposed to hypoxia (10% oxygen) and gavaged with AS‐IV (20, 40 and 80 mg/kg/day) to create a PAH model. In vitro, human pulmonary artery endothelial cells (hPAECs) were exposed to hypoxia (3% oxygen) or monocrotaline pyrrole (MCTP, 60 μg/mL) and treated with AS‐IV (10, 20 and 40 μM), EGF (10 nM, ERK agonist), small interfering CCN1 (CCN1 siRNA) and recombinant CCN1 protein (rCCN1, 100 ng/mL). We identified the differences in the expression of genes in the lung tissues of PAH rats by proteomics. At the same time, we dynamically detected the expression of CCN1 by Western blot both in vivo and in vitro. The Western blot experimental results showed that the expression of CCN1 increased in the early stage of PAH and decreased in the advanced stage of PAH. The results showed that compared with the control group, MCT‐ and hypoxia‐induced increased the hemodynamic parameters and apoptosis. AS‐IV can improve PAH, as characterized by decreased hemodynamic parameters, vascular wall area ratio (WA%), vascular wall thickness ratio (WT%) and α‐SMA expression and inhibition of cell apoptosis. Moreover, the improvement of PAH by AS‐IV was accompanied by increased CCN1 expression, which activated the ERK1/2 signalling pathway. Meanwhile, CCN1 and p‐ERK1/2 were inhibited by siCCN1 and promoted by rCCN1. EGF not only activated the ERK1/2 signalling pathway but also induced the expression of CCN1. In conclusion, AS‐IV improves PAH by increasing the expression of CCN1 and activating the ERK1/2 signalling pathway. The results of our study provide a theoretical basis for additional study on the protective effect of AS‐IV against PAH.

## 1 INTRODUCTION

Pulmonary arterial hypertension (PAH) is a destructive disease, that is characterized by a continuous increase in pulmonary artery pressure (PAP), leading to right heart failure and death.^1^, ^21^ The main pathological characteristics of PAH are pulmonary vascular remodelling, including pulmonary artery endothelial cell dysfunction, excessive proliferation of smooth muscle cells, apoptosis resistance and abnormal activation of adventitia fibroblasts.^2^, ^3^ The pathogenesis of PAH is complex; however, we know little about it. In view of the severity of this condition and a lack of current treatment options, patients with PAH urgently need a drug to slow down and treat the disease.

Cysteine rich 61 (CCN1), formerly known as CYR61 (cysteine‐rich angiogenesis inducer 61), belongs to the CCN family of stromal cell proteins.^4^, ^18^, ^22^ CCN1 plays a key role in the regulation of proliferation, differentiation, apoptosis, angiogenesis and fibrosis.^4^, ^19^ It is produced and secreted by a variety of cell types, including endothelial cells, fibroblasts and smooth muscle cells.^4^, ^20^ Recently, Lan et al. found that recombinant CCN1 can promote the proliferation of PAH pulmonary artery smooth muscle cells (hPASMCs) induced by MCT.^5^ However, Seon et al. found that recombinant CCN1 can inhibit the contraction of pulmonary vascular smooth muscle during hypoxia.^6^ The proteomics results showed that the expression of CCN1 increased in the early stage of PAH and decreased in the advanced stage of PAH. Therefore, we speculated that CCN1 is involved in PAH, but further study about the specific mechanism of CCN1 in hypoxia‐ and MCT‐induced PAH is unknown.

Extracellular signal regulated kinase (ERK) is a member of the MAP kinase family, which can be phosphorylated and activated by MEK (mitogen‐activated protein kinase/extracellular signal‐regulated kinase).^7^ The literature shows that CCN1 and integrin α6β1‐binding activates autophagy activity. After autophagy activation, its apoptotic activity is inhibited by ERK and JNK activation.^8^, ^26^ Therefore, we speculated that CCN1 participates in PAH by activating the ERK1/2 signalling pathway.

Astragaloside IV (AS‐IV) is a natural triterpene glycoside extracted from Astragalus.^9^, ^10^ It is a traditional Chinese herbal medicine. Its active component AS‐IV is widely used in the treatment of cardiovascular diseases,^40^ including anti‐myocardial hypertrophy,^11^ anti‐myocardial fibrosis,^12^ anti‐hypertension^13^ and anti‐atherosclerosis.^14^ According to the research results of our laboratory and other laboratories, AS‐IV has a protective effect against PAH.^15^, ^16^, ^17^ However, the specific role of AS‐IV and CCN1 and its associated ERK1/2 signalling pathway in PAH is unclear. Therefore, in this article we studied the effect of AS‐IV against PAH by focusing on CCN1 and its related ERK1/2 signalling pathway.

## 2 MATERIALS AND METHODS

### 2.1 Materials

AS‐IV was obtained from Nanjing Jingzhu Biotechnology Company (purity > 98% measured by HPLC, Nanjing, China). Human pulmonary artery endothelial cells were purchased from BLUEFBIO (Shanghai, China). Dimethyl sulfoxide (DMSO) was purchased from Sigma Aldrich (St. Louis, MO, United States). CCN1‐small interfering lentiviruses were purchased from Shanghai Just Science. (Shanghai, China). Recombinant CCN1 protein was purchased from R&D Systems (Minneapolis, MN). Fetal bovine serum (FBS, LOT 0001644044) was purchased from Sigma. EGF was purchased from purchased Sigma (St. Louis, MO, USA). The A5,5′,6,6′‐tetrachloro‐1,1′,3,3′‐tetraethyl‐lbenzimidazol‐carbocyanine iodide (JC‐1) kit and Fluo‐4 were obtained from the Beyotime Institute of Biotechnology (Nanjing, China). Antibodies against Bcl‐2 (A0208), Bax (A0207), Caspase‐3 (A2156), CCN1 (A7632), ERK (A16686) and p‐ERK (AP0886), and β‐actin (AC038) were purchased from ABclonal (ABclonal Technology Co.,Ltd.). A terminal deoxynucleotidyl transferase‐mediated dUTP Nick‐End Labeling (TUNEL) kit was purchased from Roche (Darmstadt, Germany).

### 2.2 Animal experiments

All animal procedures were performed under the principles approved by the Animal Ethics Committee of Jinzhou Medical University. Male Sprague Dawley (SD) rats weighing 220–250 g (obtained from the Animal Center, Jinzhou Medical University, Jinzhou, China) were allowed to adapt for one week in a controlled environment (free access to food and water), 12 h light/dark cycle and 25 ± 2°C. The rats were randomly divided into the following groups (all *n* = 10) (a) the control group (Con), (b) the hypoxia model group (Hyp, 10% oxygen), (c) the hypoxia + AS‐IV group (Hyp + AS‐IV 20 mg/kg/day), (d) the hypoxia + AS‐IV group (Hyp + AS‐IV 40 mg/kg/day) and (e) the hypoxia + AS‐IV group (Hyp + AS‐IV 80 mg/kg/day). The other groups were (a) the normal group (Nor), (b) the MCT group (MCT, 60 mg/kg), (c) the MCT + AS‐IV group (MCT + AS‐IV 20 mg/kg/day), (d) the MCT + AS‐IV group (MCT + AS‐IV 40 mg/kg/day), and (e) the MCT + AS‐IV group (MCT + AS‐IV 80 mg/kg/day). 20 mg/kg, 40 mg/kg and 80 mg/kg AS‐IV were dissolved with 0.5% sodium carboxymethylcellulose (CMC‐Na), and the amount of each gavage was 2.2–2.5 mL. SD rats in the AS‐IV group were administered 20 mg/kg, 40 mg/kg and 80 mg/kg for 28 days by gavage after hypoxia and MCT administration.

### 2.3 Hemodynamic analysis

After 28 days, the rats were anaesthetized by intraperitoneal injection of sodium pentobarbital (20%, American, Sigma). The right external jugular vein was separated, and a polyethylene 50 catheter connected to a pressure sensor was inserted into the right external jugular vein. Then, the polyethylene 50 catheter was inserted into the right ventricle and pulmonary artery. The right ventricular systolic pressure (RVSP) and mean pulmonary artery pressure (mPAP) were detected using a pressure sensor system. The ratio of the right ventricular weight to the left ventricular weight (RV) was measured RV/(LV + s) using weighing method. Finally, the SD rats were euthanized, the left lung tissue and left upper pulmonary artery were collected, and stored at −80°C until use, and the remaining lung tissue was fixed in formalin.

### 2.4 HE staining

The lung tissues of the rats were isolated. The tissue was immediately fixed in 4% paraformaldehyde for 24 h and embedded in paraffin. The tissues were cut into 5 μm sections. Haematoxylin and eosin (H&E) staining was used to detect the vascular wall area ratio (WA%) and vascular wall thickness ratio (WT%).

### 2.5 Immunohistochemical staining

After dewax and antigen repair, peroxidase activity was eliminated and nonspecific binding was blocking. The slides were then incubated overnight with primary antibodies against CCN1 (1:100) and Caspase‐3 (1:100) at 4°C and then incubated with 1:1000 anti IgG antibody at 37°C for 1 h in a moist chamber. Then, the sections were stained with DAB and haematoxylin.

### 2.6 TUNEL assay

TUNEL analysis was performed according to the manufacturer's protocol.^10^ The apoptotic index expressed the number of apoptotic cells/total number of cells × 100%.

### 2.7 Proteomics analysis

At the 4th week, lung tissue samples were collected from the control group and hypoxia‐induced PAH rats. Total protein was extracted from the lung tissues and subjected to trypsin enzymolysis, TMT labelling, HPLC classification and finally analysed by liquid chromatography‐mass spectrometry.

### 2.8 Immunofluorescence

The slides were permeabilized in PBS with 0.3% Triton X‐100 for 15 min, and then incubated in PBS with 5% bovine serum albumin for 30 min to block non‐specific binding. The slides were incubated with primary antibodies against CCN1 (1:100), Caspase‐3 (1:100) and α‐SMA (1:100) at 4°C overnight. Then, the slides were incubated with HRP secondary antibody bound to fluorescein isothiocyanate (FITC) and Hoechst 33258 staining solution.

### 2.9 JC‐1 staining

After different treatments, hPAECs cells were isolated and cultured at 37°C for 30 min in the 100 μL JC‐1 at 37°C. Then, the cells were washed twice with JC‐1 buffer.

#### 2.9.1 Cell culture

Human pulmonary artery endothelial cells (hPAECs) were purchased from BLUEFBIO (Shanghai, China). HPAECs were cultured in DMEM containing 10% FBS and 100 U/mL penicillin/streptomycin at 37°C and 5% CO_2_. HPAECs were cultured with EGF (10 nM) and rCCN1 (100 ng/mL) for 30 min, followed by AS‐IV (10, 20 and 40 μM), hypoxia chamber culture (3% oxygen) or MCTP (60 μg/mL) for 24 h. HPAECs were prepared at 5 × 10^4^/mL and infected with small interfering RNA targeting CCN1 (Shanghai, Justice Science) at multiple infection rates (MOIs) of 50, 80 and 100 at 37°C for 24 h. Then, the hPAECs cells were washed and cultured in fresh medium for 24 h for further analysis.

#### 2.9.2 CCK‐8 assay

Cell counting kit‐8 (Beyotime, Shanghai, China) was used to test cell viability according to the manufacturer's protocol. Cells were cultured in a 96‐well microplate at 10^4^ cells per well in 100 μL medium. Then, different concentrations of AS‐IV (0, 10, 20 and 40 μM) were used. The cells were treated with EGF (10 nM). After 24 h of treatment, 100 μL of CCK‐8 reagent was added to each well and then cultured for 3 h. All experiments were conducted in triplicate. Absorbance was analysed at 450 nm using a microplate reader, using a cell‐free well as a blank.

#### 2.9.3 Flow cytometry assay

Apoptosis was detected using an Annexin V‐FITC apoptosis detection kit according to the manufacturer's instructions. In short, hPAECs cells were plated at 25 cm^2^. They were incubated with the indicated drugs for 24 h. The cells were collected, washed twice with prefrozen PBS, centrifuged at 1000 rpm for 3 min and then stained with Annexin V‐FITC.

#### 2.9.4 Western blot

The collected lung tissue and hPAECs were homogenized in RIPA lysis buffer. The protein concentration was measured using a BCA protein analysis kit. The samples were separated by SDS‐PAGE (10% polyacrylamide gel) and transferred to PVDF membranes. The membrane was blocked with 1% BSA for 1 h and then incubated overnight at 4°C with antibodies against CCN1 (1:1000), Bax (1:1000), Caspase‐3 (1:1000), Bcl‐2 (1:1000) and β‐actin (1:100,000). The membrane was washed three times with TBST and then incubated with HRP (1:10,000) bound secondary antibody at room temperature for 1 h.

#### 2.9.5 Data analysis

The data are expressed as the means ± SEM and were analysed by using SPSS 25.0. The data were subjected to *t*‐tests. *p* < 0.05 was considered statistically significant.

## 3 RESULTS

### 3.1 Expression of CCN1 in pulmonary arterial hypertension

**FIGURE 1:**
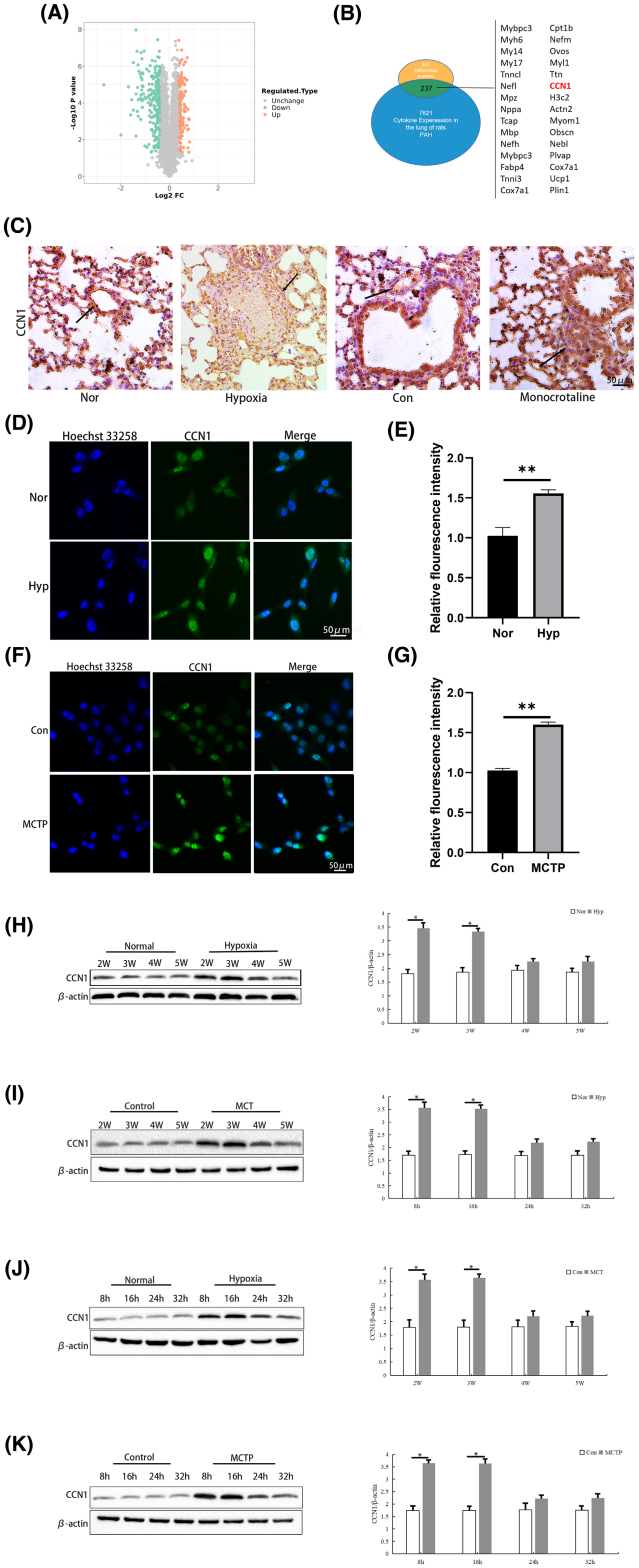
Expression of CCN1 in pulmonary arterial hypertension. (A and B) The proteomic analysis results are shown as volcano plots. (C) The expression of CCN1 in lung tissues of the control group and hypoxia‐ or MCT‐induced group was detected by immunohistochemistry. *n* = 3. (D–G) The expression of CCN1 in hPAECs of the control group and hypoxia‐ or MCTP‐ induced group was detected by immunofluorescence. *n* = 3. (H–K) Western blot was used to detect the expression of CCN1 protein in lung tissue and hPAECs under control conditions and induced by hypoxia, MCT or MCTP. *n* = 3. The data are expressed as the means ± SEM. **p < 0.01 versus the Con/Nor group, *p < 0.05 versus the Con/Nor group.

By application of protein mass spectrometry, we identified 407 differentially expressed proteins in lungs between PAH and control group rats four weeks after hypoxia treatment, including showed levels of 170 proteins were higher while those of 237 proteins were lower in PAH compared with those in control groups. We listed the differentially downregulated expressed proteins, among which, the CCN1 in PAH was decreased compared with the control group (Figure 1A,B). Next, the immunohistochemistry (Figure 1C) and immunofluorescence (Figure 1D–G) results showed that on the 28th day of hypoxia‐ and MCT‐induced PAH, the expression of CCN1 increased compared with that in the control group. To further verify the expression of CCN1, we performed Western blotting in both in vivo and in vitro studies (Figure 1H–K). The results from the in vivo study showed that CCN1 was highly expressed from the early to advanced stage of PAH (the 2nd week to the 3rd week after MCT injection or hypoxia). However, we found that the expression of CCN1 in rat lung tissues was decreased from the 4th week to the 5th week after hypoxia or MCT injection. In vitro, we also found similar changes in the expression of CCN1.

### 3.2 AS‐IV has a protective effect against pulmonary arterial hypertension

**FIGURE 2:**
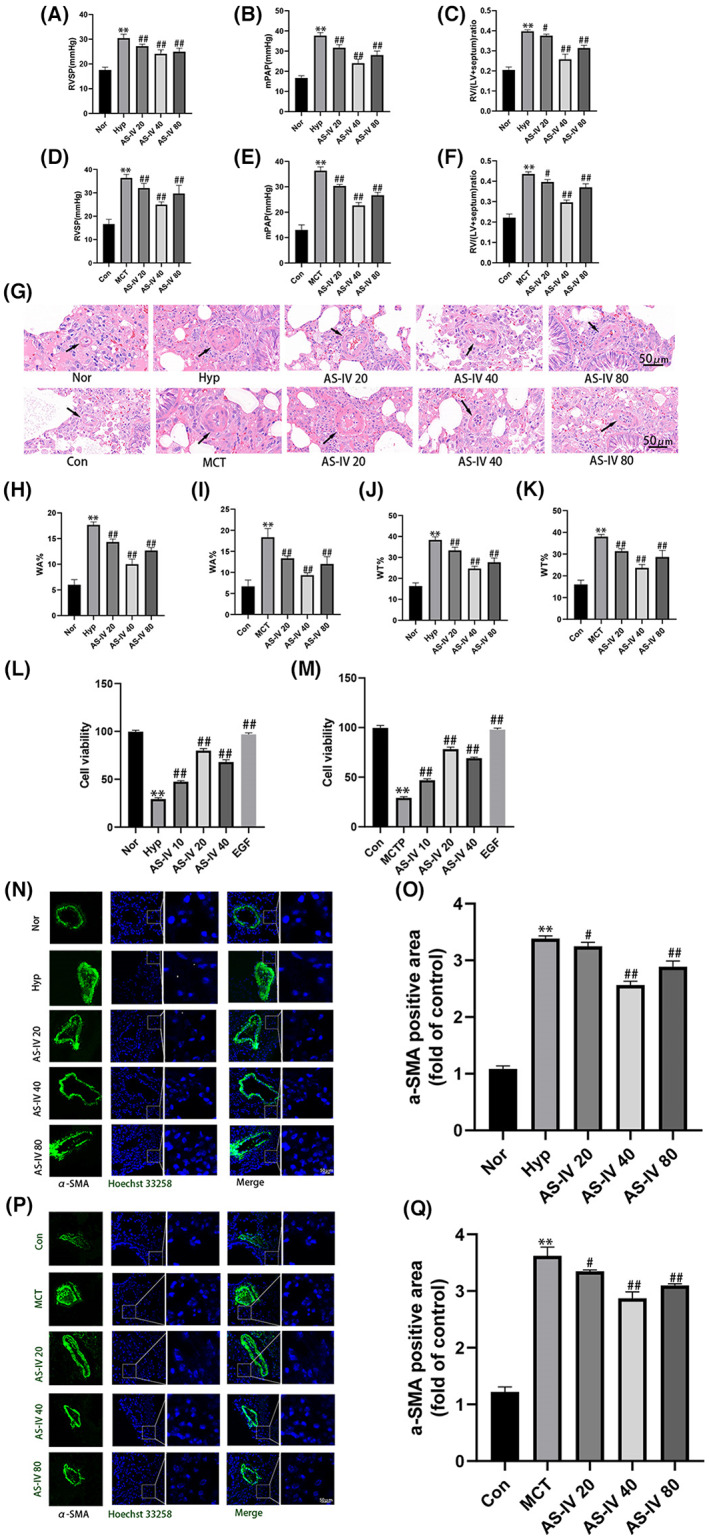
AS‐IV has a protective effect against pulmonary arterial hypertension. (A–F) Hemodynamic detection of RVSP, mPAP, RV/(LV + s), *n* = 8. (G–K) Representative images of haematoxylin eosin (H & E), *n* = 3. (L–M) CCK8 assay detection of cell viability. (N–Q) Representative images of α‐SMA immunofluorescence staining, *n* = 3. The data are expressed as the means ± SEM. **p < 0.01 versus the Con/Nor group. ^#^ *p <* 0.05, ^##^ *p <* 0.01 versus the Hyp/MCT/MCTP group.

Hemodynamic test results showed that RVSP, mPAP and RV/(LV + s) were higher in rats induced by hypoxia or MCT, indicating that PAH models had been established. Treatment with AS‐IV (20, 40 and 80 mg/kg/d) improved the above indicators (Figure 2A–F). Haematoxylin and eosin (H & E) (Figure 2G–K) and immunofluorescence staining (Figure 2N–Q) showed that after hypoxia treatment, the WA%, WT% and α‐SMA‐positive areas were significantly increased. When treated with AS‐IV (20, 40 and 80 mg/kg/day), the vascular remodelling induced by PAH was improved. Similar results were observed in MCT‐induced PAH rats. In addition, we detected cell viability by CCK‐8 assay (Figure 2L,M). The cell activity of hPAECs increased significantly after treatment with AS‐IV and EGF, indicating the therapeutic effects of AS‐IV on PAH.

### 3.3 AS‐IV can increase the expression of CCN1 in pulmonary arterial hypertension

**FIGURE 3:**
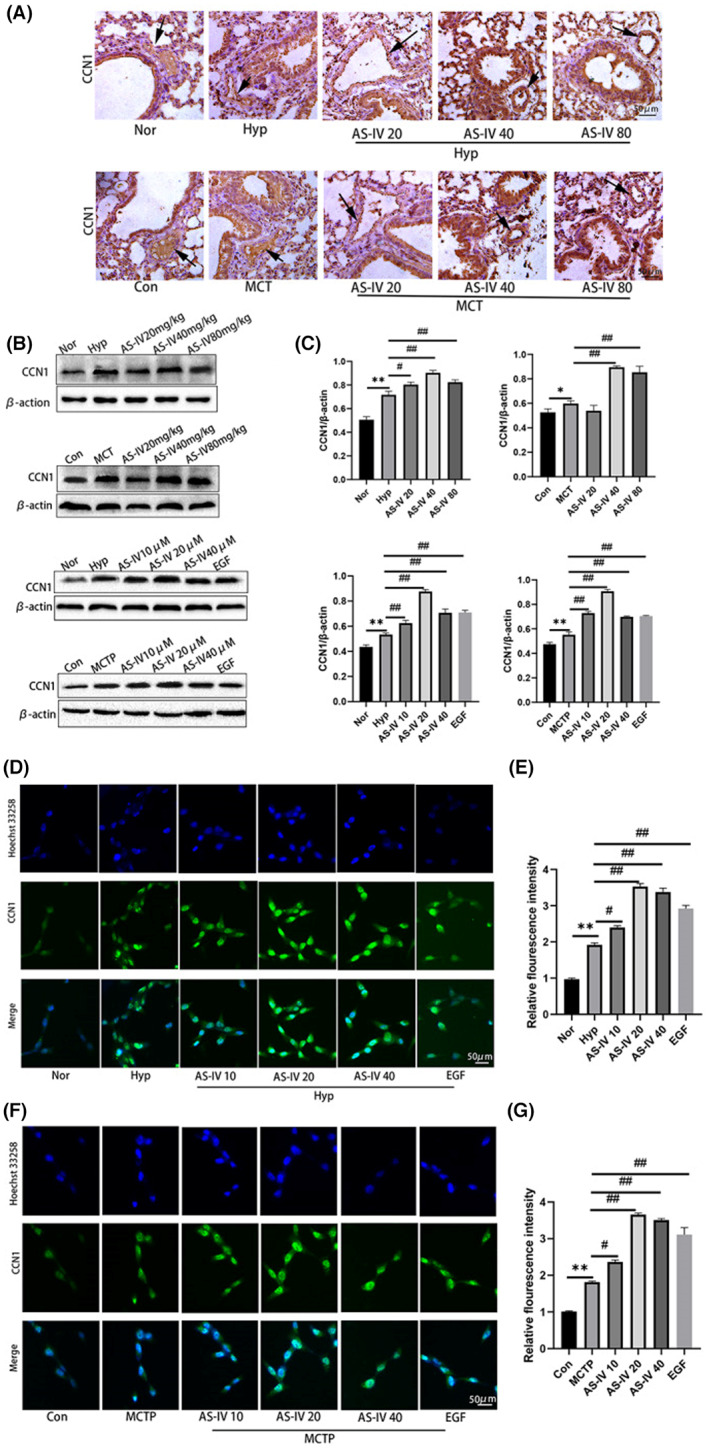
AS‐IV can increase the expression of CCN1 in pulmonary arterial hypertension. (A) The expression level of CCN1 in lung tissue was detected by immunohistochemistry. *n* = 3. (B and C) The expression level of CCN1 in lung tissue and hPAECs was detected by Western blot. (D–G) The expression level of CCN1 in hPAECs was detected by immunofluorescence. *n* = 3. The data are expressed as the means ± SEM. **p < 0.01 versus the Con/Nor group. ^#^ *p <* 0.05, ^##^ *p <* 0.01 versus the Hyp/MCT/MCTP group.

We found that CCN1 expression decreased at the 4th week and 24 h of PAH in vivo and in vitro, respectively. Thus, the 4th week and 24 h of PAH in vivo and in vitro were the time points at which the effect of AS‐IV was investigated. The expression of CCN1 was detected by immunohistochemistry (Figure 3A), immunofluorescence (Figure 3D–G) and Western blot (Figure 3B,C). At the detection point of the 4th week, the expression of CCN1 decreased, but it was still higher than that in the normal group. The administration of AS‐IV (20, 40 and 80 mg/kg/day) further increased the expression of CCN1 in both hypoxia‐ and MCT‐induced PAH. The in vitro results were consistent with those in vivo, and EGF showed a similar effect to AS‐IV.

### 3.4 AS‐IV inhibited cell apoptosis

**FIGURE 4:**
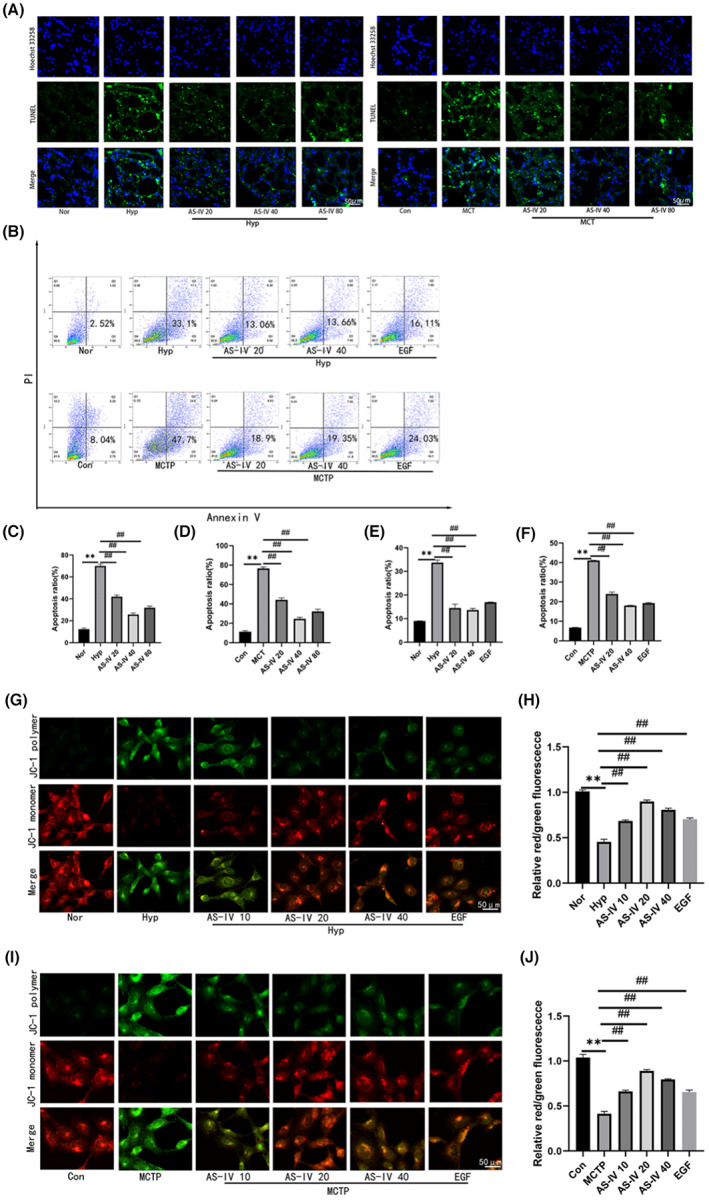
AS‐IV inhibited cell apoptosis. (A,C,D) Apoptosis was detected by TUNEL fluorescence staining. (B,E,F) The apoptosis level of hPAECs cells was detected by Annexin‐V/PI staining and flow cytometry. The upper and lower right regions were regarded as apoptotic regions. (G–J) HPAECs cells were stained with JC‐1, and the mitochondrial membrane potential was evaluated by fluorescence microscopy. The data are expressed as the means ± SEM, *n* = 3. **p < 0.01 versus the Con/Nor group. ^#^ *p <* 0.05, ^##^ *p <* 0.01 versus the Hyp/MCT/MCTP group.

Both in vivo and in vitro, we found that AS‐IV increased the expression of CCN1. Next, we explored the relationship between increased CCN1 and apoptosis caused by PAH. TUNEL staining (Figure 4A,C,D) was used as an apoptosis assay in vivo, and JC‐1 (Figure 4G–J) and flow cytometry (Figure 4B,E,F) were used as apoptosis assays in vitro. The results showed that compared with hypoxia or MCTP group, AS‐IV treatment inhibited cell apoptosis both in vivo and in vitro.

### 3.5 AS‐IV regulates cell apoptotic genes

**FIGURE 5:**
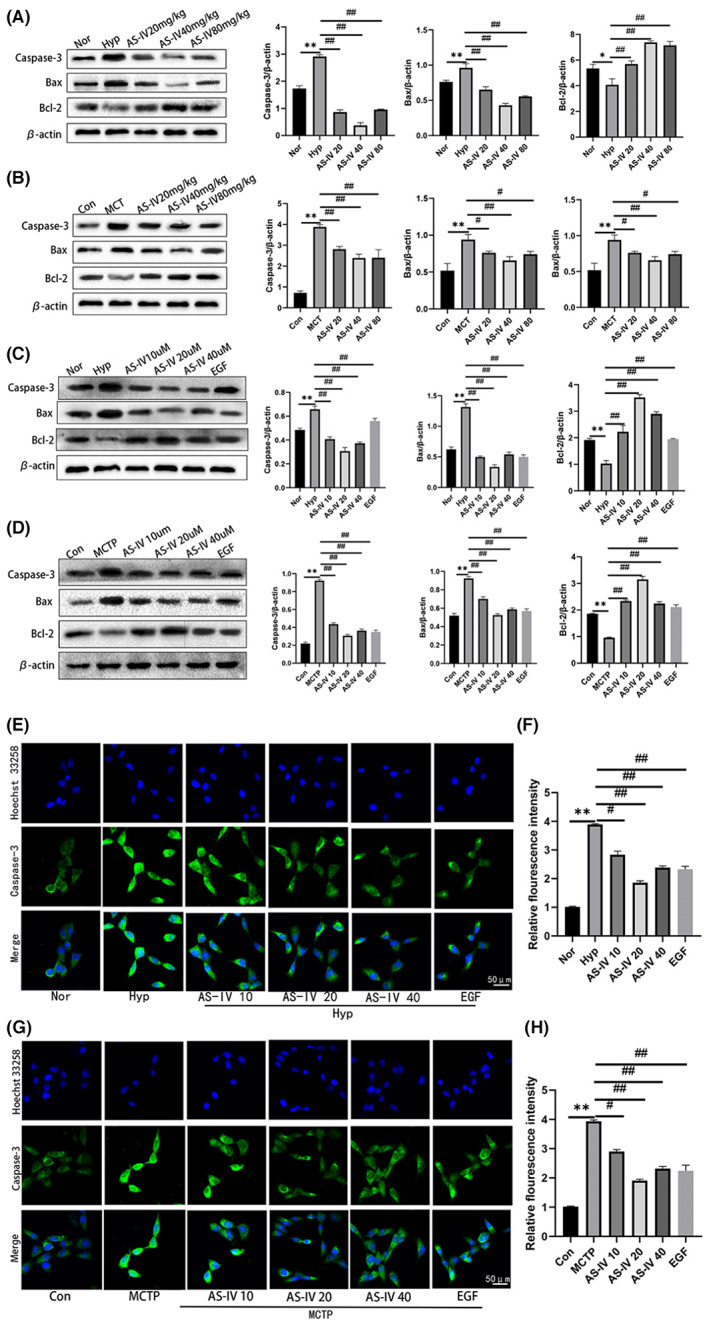
AS‐IV regulates cell apoptotic genes. (A–D) Western blot showing the expression levels of Caspase‐3, Bax and Bcl‐2 in lung tissues or hPAECs. (E–H) The expression of Caspase‐3 in hPAECs was detected by immunofluorescence. The data are expressed as the means ± SEM, *n* = 3. **p < 0.01 versus the Con/Nor group. ^#^ *p <* 0.05, ^##^ *p <* 0.01 versus the Hyp/MCT/MCTP group.

To investigate the antiapoptotic effect of AS‐IV on PAH further, the expression of apoptotic genes were measured. The results showed that compared with hypoxia or MCT, AS‐IV treatment decreased Bax and Caspase‐3 expression, and increased Bcl‐2 expression both in vivo and in vitro (Figure 5). Combined with the abovementioned results, these findings suggest that AS‐IV could attenuate cell apoptosis induced by hypoxia and MCT.

### 3.6 CCN1 regulates ERK1/2 signalling

**FIGURE 6:**
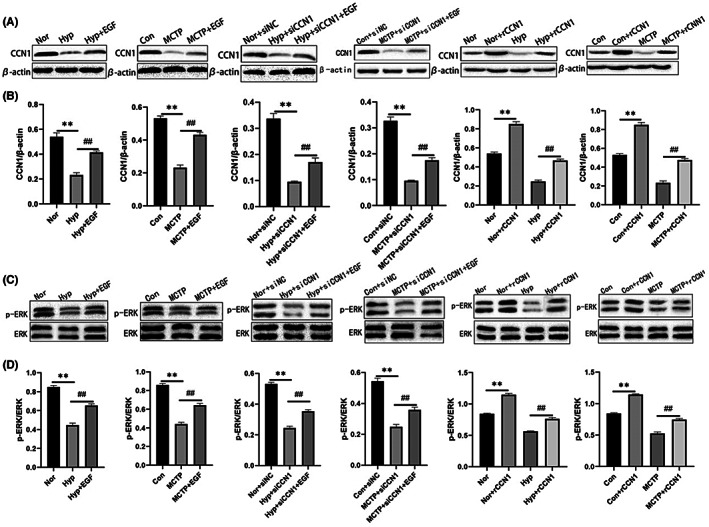
CCN1 Regulates ERK1/2 signalling. (A–D) The expression levels of CCN1, ERK and p‐ERK proteins in hPAECs treated with siCCN1, rCCN1 and EGF were detected by Western blot. The data are expressed as the means ± SEM, *n* = 3. **p < 0.01 versus the Con/Nor group. ^#^ *p <* 0.05, ^##^ *p <* 0.01 versus the Hyp/MCTP group.

To investigate the relationship between CCN1 and ERK1/2, we used small interferences of CCN1 (siCCN1), recombinant CCN1 protein (rCCN1), and EGF (an agonist of ERK) to detect the effects of CCN1 on ERK1/2. The results showed that the expression of CCN1 and p‐ERK1/2 was inhibited by siCCN1, and the expression of CCN1 and p‐ERK1/2 was promoted by rCCN1. Moreover, EGF, an agonist of ERK1/2, not only activated the ERK1/2 signalling pathway but also induced the expression of CCN1. These results demonstrated that there was crosstalk between CCN1 and the ERK1/2 signalling pathway (Figure 6).

## 4 DISCUSSION

PAH is a chronic obstructive pulmonary disease that eventually leads to heart failure and death and currently, cannot be cured. This feature makes PAH a devastating diagnosis.^23^, ^24^, ^25^ Therefore, patients with PAH take various medications to alleviate symptoms. AS‐IV is the main component of *Astragalus membranaceus* and it has protective effects on the cardiovascular system, immune system and nervous system. According to the research results of our laboratory and other laboratories, AS‐IV has a protective effect against PAH. In our study, PAH models were established both in vivo and in vitro. The results of hemodynamics, H&E, α‐SMA immunofluorescence staining and CCK‐8 cell activity assays showed that AS‐IV had a protective effect against PAH. The results of TUNEL fluorescence detection, JC‐1, immunofluorescence, immunohistochemistry, flow cytometry and Western blotting showed that AS‐IV could alleviate the apoptosis caused by PAH and reduce the expression of apoptosis‐related genes. This further shows that AS‐IV has a protective effect against PAH.

Proteomics essentially refers to the study of protein characteristics at a large‐scale level, including the protein expression level, post‐translational modification and protein–protein interactions, to obtain an overall and comprehensive understanding of disease occurrence, cell metabolism and other processes at the protein level. Therefore, we performed proteomics analysis to detect the expression level of genes in lung tissue at the 4th week of PAH‐induced hypoxia. Through the model, we found that compared with the control group, the expression of CCN1 decreased in the advanced stage of PAH with high specificity. Therefore, we chose CCN1 as our target protein for further study.

CCN1 is one of the main members of the extracellular matrix protein CCN family. CCN1 is produced and secreted by smooth muscle cells, endothelial cells and fibroblasts. CCN1 plays a key role in the regulation of proliferation, differentiation, apoptosis, angiogenesis and fibrosis.^4^ CCN1 is expressed in a variety of lung diseases, but its specific role is controversial. In a model of acute lung injury (ALI), studies have shown that CCN1 overexpression activates Akt and downstream signalling pathways, promotes cell proliferation and protects lung epithelial cells from apoptosis induced by hyperoxia.^27^, ^28^ However, the study of Grazioli et al. showed that adenovirus over‐expressing CCN1 enhanced lung inflammation.^29^ Obviously, these effects of CCN1 are different in the same lung disease model. In a model of bronchopulmonary dysplasia (BPD), neonatal rats were treated with recombinant CCN1 protein, and it reduced hyperoxia‐induced lung injury, indicating that CCN1 has an anti‐inflammatory effect.^30^ Cigarette smoking (CS) is the main risk factor for chronic obstructive pulmonary disease (COPD).^31^, ^32^ In a model of COPD, CS induces the activation of reactive oxygen species (ROS) in the endoplasmic reticulum (ER), increases the expression of CCN1 in alveolar epithelial cells and activates the Wnt signalling pathway, triggering the release of IL‐8.^33^ In a pulmonary fibrosis model, Kurundkar et al. showed that CCN1 was upregulated in the lung tissue of patients with idiopathic pulmonary fibrosis and it activated the TGF‐β1/Smad3 signalling pathway, CCN1 was expressed in response to the increase in the expression of profibrotic genes induced by the lung injury, resulting in pulmonary fibrosis.^34^, ^35^ In a lung infection model, the virulence factor. A toxin of *Staphylococcus aureus* significantly inhibited the expression of CCN1 in alveolar macrophages (AMs), resulting in a decrease in the ability of AMs to scavenge neutrophils.^36^, ^37^ In a lung cancer model, researchers have confirmed that the CCN1 protein is involved in the occurrence of non‐small cell lung cancer, and further showed that the CCN1 plays a tumour inhibitory role in non‐small cell lung cancer.^38^, ^39^ In conclusion, the effect of CCN1 is different in different lung disease models.

In our study, the Western blot results showed that compared with the control group, the expression of CCN1 increased significantly from the early to the advanced stage of PAH, and over time, the increase of CCN1 showed a downward trend, but it was still higher than that in the control group. Similar to this phenomenon, Lan et al.^5^ observed that a large number of inflammatory factors are released in the early stage of PAH, PAH induces the expression of CCN1 while promoting inflammatory stimulation, creating a positive feedback cycle for the production of more CCN1. In the advanced stage of PAH, the release of inflammatory factors decreases and fibrosis gradually worsens, accompanied a decrease in CCN1 expression. However, some studies have shown that the production and secretion of CCN1 vary with the time of modelling, dosage and measurement methods. Therefore, the specific mechanism regulating the expression of CCN1 needs further research and discussion.

Based on our Western blot results, we decided to conduct further experiments at two time points: 28 days in vivo and 24 h in vitro. Our study found that AS‐IV could increase the expression of CCN1 both in vivo and in vitro, which was verified by immunohistochemistry, immunofluorescence and Western blotting. According to the results of TUNEL, flow cytometry, JC‐1, immunofluorescence and Western blotting, the upregulating effect of AS‐IV on CCN1 was accompanied by a reduction in the cell apoptosis caused by PAH. These results suggested that AS‐IV has a protective effect against PAH by upregulating CCN1.

When discussing the relationship between CCN1 and ERK1/2, we applied small interferences of CCN1 (siCCN1), recombinant CCN1 protein (rCCN1) and EGF (an agonist of ERK). Western blotting results demonstrated that siCCN1 could knock down the expression of CCN1 and inhibit the activity of ERK1/2. RCCN1 could increase the expression of CCN1 and further activate the activity of ERK1/2. EGF could not only activate the activity of ERK1/2 but also induce the expression of CCN1. These results suggested that there is an interaction between CCN1 and ERK1/2 in hypoxia and MCT‐induced PAH. Therefore, we concluded that AS‐IV could increase the expression of CCN1 in the advanced state of PAH, activate ERK1/2 signalling pathway, reduce apoptosis caused by PAH and ameliorate PAH, which provides a theoretical basis for further study of protective effects of AS‐IV against PAH.

## AUTHOR CONTRIBUTIONS

**Yu Liu:** Writing – original draft (equal). **Bai‐Lin Tang:** Data curation (equal); methodology (equal); software (equal). **Mei‐Li Lu:** Writing – review and editing (equal). **Hong‐Xin Wang:** Funding acquisition (supporting).

## CONFLICT OF INTEREST STATEMENT

All authors claimed that there was no conflict of interest in the study.

## Data Availability

The data used to support the findings of this study are available from the corresponding author upon request.
